# DREAM3: Network Inference Using Dynamic Context Likelihood of Relatedness and the Inferelator

**DOI:** 10.1371/journal.pone.0009803

**Published:** 2010-03-22

**Authors:** Aviv Madar, Alex Greenfield, Eric Vanden-Eijnden, Richard Bonneau

**Affiliations:** 1 Center for Genomics and Systems Biology, Department of Biology, New York University, New York, New York, United States of America; 2 Computational Biology Program, New York University School of Medicine, New York, New York, United States of America; 3 Department of Mathematics, Courant Institute of Mathematical Sciences, New York University, New York, New York, United States of America; 4 Department of Computer Science, Courant Institute of Mathematical Sciences, New York University, New York, New York, United States of America; Center for Genomic Regulation, Spain

## Abstract

**Background:**

Many current works aiming to learn regulatory networks from systems biology data must balance model complexity with respect to data availability and quality. Methods that learn regulatory associations based on unit-less metrics, such as Mutual Information, are attractive in that they scale well and reduce the number of free parameters (model complexity) per interaction to a minimum. In contrast, methods for learning regulatory networks based on explicit dynamical models are more complex and scale less gracefully, but are attractive as they may allow direct prediction of transcriptional dynamics and resolve the directionality of many regulatory interactions.

**Methodology:**

We aim to investigate whether scalable information based methods (like the Context Likelihood of Relatedness method) and more explicit dynamical models (like Inferelator 1.0) prove synergistic when combined. We test a pipeline where a novel modification of the Context Likelihood of Relatedness (mixed-CLR, modified to use time series data) is first used to define likely regulatory interactions and then Inferelator 1.0 is used for final model selection and to build an explicit dynamical model.

**Conclusions/Significance:**

Our method ranked 2nd out of 22 in the DREAM3 100-gene *in silico* networks challenge. Mixed-CLR and Inferelator 1.0 are complementary, demonstrating a large performance gain relative to any single tested method, with precision being especially high at low recall values. Partitioning the provided data set into four groups (knock-down, knock-out, time-series, and combined) revealed that using comprehensive knock-out data alone provides optimal performance. Inferelator 1.0 proved particularly powerful at resolving the directionality of regulatory interactions, i.e. “who regulates who” (approximately 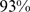 of identified true positives were correctly resolved). Performance drops for high in-degree genes, i.e. as the number of regulators per target gene increases, but not with out-degree, i.e. performance is not affected by the presence of regulatory hubs.

## Introduction

For decades the biological community has had a keen interest in characterizing the genetic regulatory networks that are largely responsible for an organisms ability to adapt to its constantly changing environment. An ever increasing number of functional genomics projects continue to make this a key problem in modern biology. It remains, however, unclear what constitutes the most efficient paradigm for characterizing regulatory networks, i.e. what experiments to perform, data to collect, and methods to use for learning biological regulatory networks. Moreover, the number of proposed methods for learning regulatory networks from systems data is growing and it is difficult to compare the relative merit of these methods unless methods are evaluated on similar datasets using similar metrics. The DREAM (Dialogue for Reverse Engineering Assessments and Methods) project [1], [2] aims to shed light on which paradigm is most useful for characterizing regulatory networks. It does so by posing a set of challenges to the computational biology community at large, allowing for the comparison of different methods on identical footing.

There are several broad classes of regulatory network inference methods that aim to reconstruct and model the underlying regulatory networks at varying degrees of detail. It is beyond the scope of this introduction to review more than a small subset of these methods, as they represent a very large body of work, for a more thorough review of network reconstruction methods we refer the reader to [3]–[10]. Here we focus on two classes of methods, namely: Mutual Information (MI) and Ordinary Differential Equation (ODE) based methods.

Mutual information based methods [11]–[17] are often formulated such that it is not necessary to assume a functional form for the effect of a regulator on its target(s); mutual information does not assume a linear relationship between any given pair of genes (or any parametric relationship, for that matter). These methods often scale well to genome-wide regulatory networks, providing advantages over more detailed models in cases where the functional forms of regulatory interactions are unknown, complex, or when there is insufficient data to learn more intricate models. However, MI based methods as previously formulated, provide limited insight into the dynamic behavior of the system, and hence have limited use in predicting new observations—a key property for estimating a model's relevance when the ground truth is unknown.

Ordinary Differential Equation based methods [18]–[30], aim to learn a set of ODEs describing the time evolution of target genes as a function of their likely regulators. These methods can provide deeper understanding of the system's dynamic behavior, and can be used to predict new observations. However, they can be computationally demanding, and may require accurate measurement of a large number of parameters.

Here we employ a modified version of the MI based method Context Likelihood of Relatedness (CLR) [14] (modified to use time-series data) to reduce the space of possible regulatory interactions for a more detailed, scalable, ODE based method, Inferelator 1.0 [25]. CLR is an easily scalable MI based method for network reconstruction. In order to identify regulatory interactions CLR computes the MI between the expression levels of every possible regulator-target gene pair, and then computes a score for each pair. The score for a pair is a function of two z-scores resulting from comparing the pair's MI value with: all MI values involving the regulator (to generate the regulator z-score), and all MI values involving the target (to generate the target z-score). CLR takes advantage of the fact that biological networks are, on average, quite sparse and assumes the majority of MI values involving a given target or regulator are insignificant, and thus constitute a background MI distribution. The method has been successfully used in the past to learn previously validated, as well as novel, transcriptional regulatory interactions in *E. coli* [14]. This method, as originally published, cannot resolve causality, i.e. which gene is the regulator and which gene is the target for a given significant interaction, as it relies solely on the symmetric MI matrix and can not take advantage of the kinetics represented in time series data.

The Inferelator 1.0 is a scalable method that uses an additive ODE model to approximate regulatory dynamics. At the core of the method is an 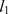-norm constrained regression algorithm, LASSO [31] (implemented using Least Angle Regression (LARS) [32]), that is used to efficiently choose a parsimonious set of likely regulators for each target gene, and to estimate the kinetic parameters associated with these interactions. Inferelator 1.0, like CLR, takes account of the sparsity found in biological networks by imposing an 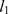 constraint on the kinetic parameters, resulting in a sparse (parsimonious) regulatory model. The method has been used successfully in the past to learn a large portion of *H. salinarum* transcriptional regulatory network, and was able to predict mRNA levels of 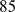 percent of the genes in the genome over new experimental conditions [33]. Two similar methods have also been successfully applied to the learning of human regulatory networks mediating TLR-5 response in macrophages [34], and to the DREAM2 50-gene *in silico* network challenge [27]. The Inferelator 1.0 as originally published included interactions between regulators in the ODE model.

Several network reconstruction methods, including the method described here, restrict the number of considered regulatory interactions using a correlation or MI based pre-processing step. For example, the Sparse Candidate Algorithm, a Bayesian network approach for learning biological regulatory networks, employed a mutual information pre-processing step, aimed at reducing complexity and improving the scaling of the algorithm to the genome scale [35]. Here, we describe and test an overall pipeline in which a modified version of CLR, a version that computes dynamic and static mutual information values for regulator-target pairs (mixed-CLR), is used as a pre-processing step for a more detailed ODE based method—Inferelator 1.0. We show that this overall dynamic pipeline identifies more directed true regulatory interactions when compared to pipelines that are based on a static model.

## Methods

**Figure 1:**
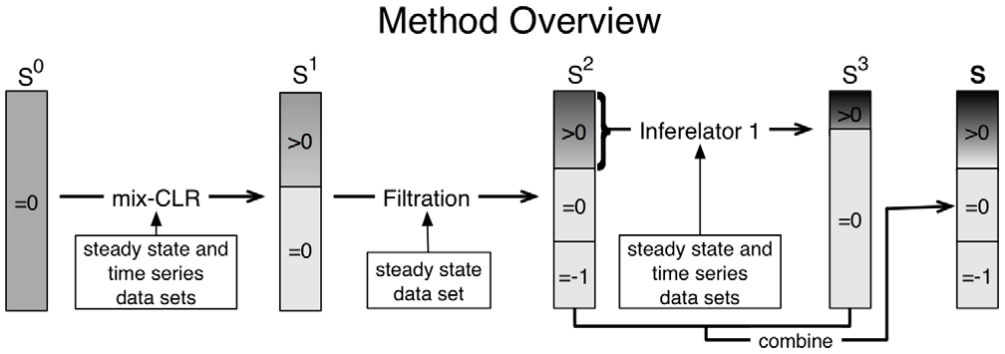
Method outline. For each regulatory interaction, 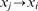, we define a confidence score 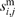, where 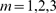 indicates the step in our pipeline. We store these confidence scores in a corresponding 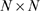 matrix, 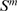 (eq. 2), which we depict in the figure as a sorted list (from high to low confidence) of regulatory interactions. We schematically represent true positives (TPs) density (within any subset) as a gray scale, where black indicates high TP density. All possible pair-wise regulatory interactions are first scored using mixed-CLR, resulting in a matrix 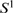. We then filter out the least likely regulatory interactions based on the knock-out and knock-down steady-state observations, resulting in a matrix 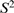 (the confidence score of each removed regulatory interaction was set to minus one, and thus sent to the back of the list). Lastly, we evaluate regulatory interactions in the TP enriched subset using Inferelator 1.0, by building an ODE model for each target gene. The kinetic weights from these ODE models were converted into confidence scores (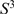) and combined with 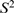 to produce the final ranked list, 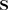 (eq. 32). The regulatory interactions scored in 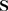, when ranked from high to low, represent our final ranking for each regulatory interaction.

Here we describe the three step pipeline (Figure 1) that we have applied to the DREAM3 in-silico network challenge [1], [2].

### Problem Set Up

The dynamical variables available from observations are the simulated mRNA levels of genes:
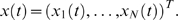
(1)


We are given data sets that contain observations taken from five different networks [36]. Each data set is composed of multiple sets of time series observations—where the system was perturbed and then measured at equal time intervals—and steady state observations—where the system was perturbed and then measured once it reached a steady state. Perturbations for time series observation consist of changing the initial expression levels of all genes. Perturbations for steady state observation consist of either knocking out one gene at a time, i.e. one of the gene's initial expression level is set close to zero, or knocking down one gene at a time, i.e. one of the gene's initial expression level is set close to half its wild-type expression level.

The DREAM3 *in silico* network challenge required participants to produce a ranked list of all possible pair-wise regulatory interactions, 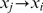 (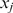 regulates 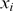), ordered by confidence. A perfect ranking would have all of the true regulatory interactions, i.e. true positives (TPs), ranked before all of the false regulatory interactions, i.e. true negatives (TNs). This paper does not address the relative strengths of the regulatory interactions or the kinetic constants learned by Inferelator 1.0, as predicting topology of a regulatory network was the main focus of this challenge.

To determine rankings, we will define a confidence score, 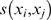, at each step in our pipeline to indicate our confidence in any given regulatory interaction, 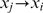. We store these values in the form of a 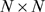 matrix of confidence scores:
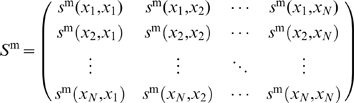
(2)where the superscript, 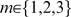, indicates our measure of confidence after steps, one, two, and three in our pipeline, respectively. Note that columns in 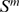 correspond to regulators, and rows correspond to targets.

Without loss of generality we can assume that time-series observations resulted from one perturbation experiment, i.e. we can write them in the form of a 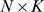 matrix of observations
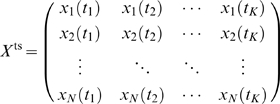
(3)where 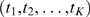 are the observation times.

There are two main sets of steady-state experiments: measurements of all genes when one gene (per experiment), 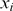, is knocked out, which we denote as 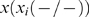; and measurements of all genes when one gene (per experiment), 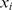, is knocked down, which we denote as 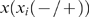. For diploid cells, cells that contain two sets of chromosomes (one set donated from each parent), the notations 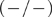 and 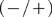 are often used to indicate that both copies of a gene are non-functional or that one copy of a gene is non-functional, respectively. These two sets of experiments are complemented with one steady-state experiment that describe the system at the lack of any perturbation, so called wild type expression levels, which we denote as 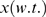.

Denote by 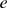 the vector of all steady-state experiments, i.e.
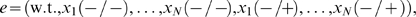
(4)then we can write all of the steady-state observations in the form of a 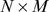 matrix
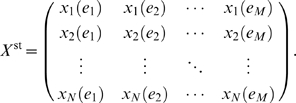
(5)where 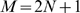 indicates the total number of steady-state experiments.

Note that unlike typical genome-wide mRNA observations, the observations given in DREAM3 ranged from zero to one (e.g. microarray and RNA-seq can exhibit multiple 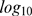 units of range). This suggested to us that the DREAM3 data set, as provided, was properly normalized and thus we did not take any further data-normalization steps.

### Step 1.a: Computing static and dynamic Mutual Information between Regulators and Targets

As the first step in our pipeline we apply our modified CLR algorithm (mixed-CLR) to reduce the number of likely regulators for each target (i.e. gene). This procedure has two parts: 1) computing static and dynamic Mutual Information (MI) between each potential regulator and target pair, followed by 2) a background correction step, for which we use the procedure originally described in [14].

We use MI as a metric of statistical dependency between two genes. MI between two random variables 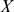 and 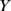 can be defined as [37], [38]

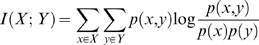
(6)where 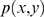 is the joint probability distribution function of 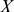 and 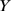, and 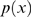 and 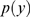 are the marginal probability distribution functions of 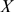 and 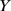, respectively, i.e. the probability that 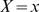 and 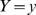, respectively.

When computing MI from continuous data a binning approach is often used [11]. Binning can lead to crude estimates of the probabilities involved, especially for small data sets. Fuzzy binning (smoothing), where each point is assigned to a number of bins with an associated weight, can alleviate this situation, leading to better estimates of probabilities. Here, we compute mutual information using a smoothing B-spline approach proposed by [39], with ten bins, and third-order B-splines (for a detailed description we refer the reader to [39]). An R [40] package for this method is available from the authors upon request (code is based in part on [14], [39]).

Using both time-series and steady-state observations (the full set of provided experiments) we compute the static MI between the observed expression levels of every gene pair, 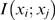, and store their values in the form of a 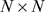 matrix,
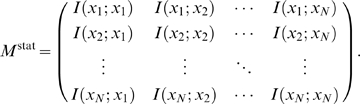
(7)


Computing MI between the expression levels of genes with the purpose of characterizing regulatory interactions has two major limitations: 1) a pair of genes can often have a high MI value due to many reasons other than a regulatory interaction, e.g. a pair of genes can share a regulator; and 2) MI between the expression levels of two genes is a symmetric quantity, and thus can not resolve causality, i.e. can not resolve the directionality of the regulatory interaction. To partially resolve these limitations we compute dynamic MI values, derived from a linear additive ODE model, motivated by our previous work on Inferelator 1.0 [25].

We assume that the time evolution in the 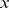
's can be approximated by the linear ODE:
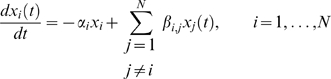
(8)where 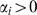 is the first-order degradation rate of 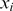, and
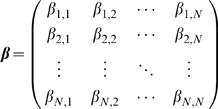
(9)is a set of parameters to be estimated. Note that the matrix 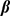 is typically sparse, i.e. most entries are 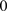, and that it is given that auto-regulatory interactions do not exist in any of the DREAM3 networks, i.e. 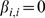 for all 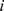.

The next two steps aim to separate the terms in (8) that involve the putative regulators (i.e. the explanatory variables) from the terms in (8) that involve the target (i.e. the response), first for time-series experiments and then for steady-state experiments.

For time-series experiments we can write (8) using a finite difference approximation as
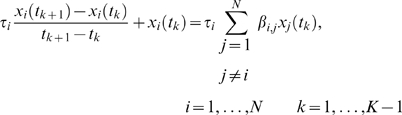
(10)where 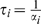 is related to the half-life of 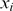 by 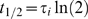, and is set throughout this work to 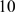 minutes (i.e. half-life time of 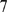 minutes). This value is in the range of many known mRNA half-life times for *E. coli* [41], and previous work has shown that error in 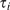 can be compensated for via an overall scaling of 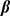 (Bonneau et al., unpublished). Thus, for every regulator (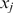) target (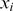) pair we can define a time-series response variable, 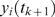, as
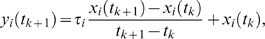
(11)with a corresponding explanatory variable, 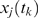; both derived from the left- and right-hand-sides of (10), respectively.

For steady state experiments we can write (8) by setting the derivative to zero as
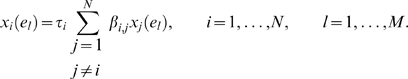
(12)Thus, for every regulator (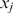) target (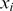) pair we can define a steady-state response variable, 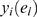, as
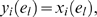
(13)with a corresponding explanatory variable, 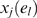; both derived from the left and right-hand-sides of (12), respectively.

Combining the time-series and steady-state response variables, we get the response vector:
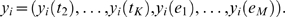
(14)


Combining the corresponding time-series and steady-state explanatory variables together, we get the explanatory variables vector:
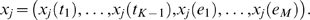
(15)Note that for time-series, each explanatory variable (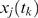) is time-lagged with respect to its corresponding response variable (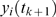). Given well-sampled time series, it is easy to see how this may help resolve causation. However, we also consider the MI between the pair 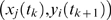 helpful at reducing statistical dependencies that are not due to direct regulatory interactions (when compared to the MI between the pair 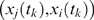). This is based on the simple, yet biologically relevant, assertion that a transcription factor (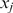) directly affects the rate of change of its target mRNA (approximated by 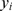) and not the accumulated amount of that target gene mRNA. In the Result section we shall see that for DREAM3 100-gene networks this is indeed the case.

We compute the dynamic MI between every pair of response-vector and explanatory-variable vector, 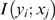, and store their values in the form of a 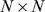 matrix,
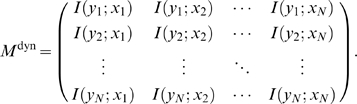
(16)


Note that static- and dynamic-MI values are estimated using the same number of observations. Next we describe how to use dynamic MI values as part of a modified CLR background correction.

#### Step 1.b: Context Likelihood of Relatedness (CLR)

At the core of both the original CLR method and our modified CLR variant, mixed-CLR, is a background correction step that computes the significance of a given regulator-target MI value by comparing that value to all MI values for that regulator and all MI values for the given target. This background correction step can be briefly described as follows:

Let 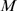 be a 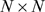 matrix, with each entry, 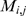, equals the pair-wise MI between a pair of variables, 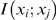. In order to derive a CLR score for that pair of variables, 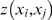, first compute a positive Z-score for 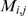 with respect to the entries in the 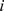
'th row of 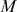, i.e.
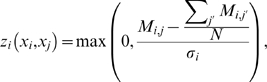
(17)where 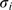 is the standard deviation of the entries in the 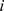
'th row of 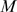. Second, compute a positive Z-score for 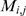 with respect to the entries in the 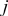
'th column of 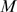, i.e.
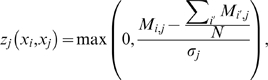
(18)where 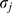 is the standard deviation of the entries in the 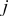
'th column of 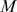. Lastly, combine the previous two positive Z-scores into a CLR pseudo Z-score, as:
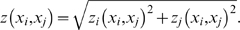
(19)


We have computed the pseudo z-scores in three variations:

CLR: We have applied CLR background correction to 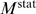 (7), resulting in a 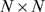 matrix of CLR pseudo Z-scores,
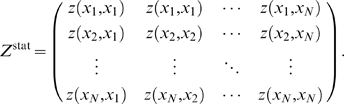
(20)


dynamic-CLR: We have applied CLR background correction to 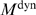 (16), resulting in a 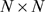 matrix of dynamic-CLR pseudo Z-scores,
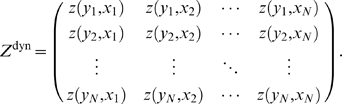
(21)


Mixed-CLR, using dynamic and static MI values: Here, we propose and describe a CLR background correction that is based in part on 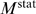, and in part on 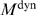. The motivation to use dynamic MI values is that they may be more appropriate to resolve true regulatory interactions from spurious dependencies. Although dynamic MI values may reduce false dependencies in the data, they will not completely remove them. The expected distribution of false or indirect dependencies is best represented by the static MI values. For these reasons we decided to evaluate a mixed (dynamic 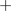 static) CLR procedure.

To apply this procedure we first, as was done for dynamic-CLR above, compute the Z-score of 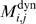 with respect to the entries in the 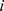
'th row of 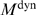, i.e.
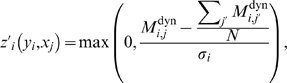
(22)where 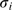 is the standard deviation of the entries in the 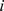
'th column of 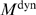.

Second, we compute the Z-scores of 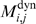 with respect to the background distribution of MI entries in the 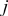
'th column of 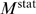, i.e.
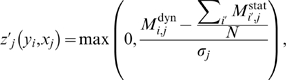
(23)where 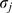 is the standard deviation of the entries in the 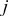
'th column of 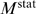. Note that 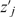 compares the dynamic MI value with the observed distribution of static MI values, and in order for this background correction step to be effective, it assumes that both dynamic and static MI values are in the same range (this was a wrong assumption as later we show that, at least for the DREAM3 100-gene networks, static MI values are in general larger then dynamic MI values).

Lastly, we combine the previous two Z-scores into a pseudo Z-score, 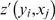, as described in (19), resulting in a 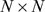 matrix of mixed-CLR Z-scores,
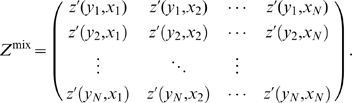
(24)


Note that 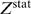 is symmetric, and thus can not be used to resolve directionality of regulatory interactions, while 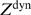, and 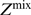 are not symmetric.

In order to decide which CLR variant to use for DREAM3 predictions, we evaluated the performance of all three CLR variants, on the two DREAM2 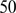-gene networks, applying the top method from this test to the DREAM3 challenge. Mixed-CLR outperformed both CLR and dynamic-CLR. Thus, our matrix of confidence scores after step one is:
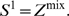
(25)The regulatory interactions scored in 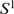, when ranked from high to low, represent our ranking for the regulatory interaction list after step one. Note, 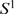 sets the confidence scores of many regulatory interactions to zero, thus removing them from consideration, while ranking the remaining interactions.

### Step 2: Using Genetic Perturbations to Remove Least Likely Regulatory Interactions from Consideration

For our second step we perform crude filtration to remove the most unlikely regulatory interactions given our knowledge of gene knock-outs and knock-downs. This step is solely based on the genetic perturbations collected as steady-state observations.

For each interaction, 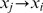, we compute the relative change in mRNA level of 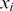 when 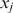 is knocked out:
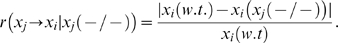
(26)Similarly, we compute the relative change in mRNA level of 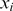 when 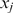 is knocked down:
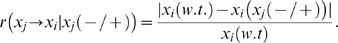
(27)Given a cutoff, 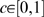, we filter out an interaction, 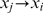, iff 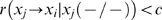 AND 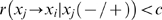. In other words, we filter out a regulatory interaction, 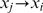, if a large drop in expression levels of 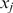 have only resulted in a smaller than 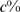 change in expression levels of 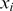. For every regulatory interaction, 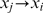, that was filtered this way, we have set 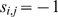. The actual value of 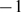 does not matter, as negative scores are sent to the end of the ranked regulatory interaction list and not considered further. We denote the matrix of confidence scores, 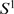, after applying filtration as 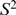.

The regulatory interactions scored in 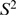, when ranked from high to low, represent our ranking for the regulatory interaction list after step two. We now apply the final step of our procedure, an ODE-based constrained linear regression approach—Inferelator 1.0.

### Step 3.a: Inferelator 1.0

Here we use the results of the previous two steps, contained in 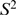, to remove low ranked regulatory interactions from consideration by Inferelator 1.0 [25], improving overall model selection performance. Furthermore, we want to force Inferelator 1.0 to consider only high confidence regulatory interactions (i.e. high rank regulatory interactions), strengthening the connection between mix-CLR and Inferelator 1.0. Thus, as possible regulators (explanatory variables) of 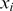, we consider the 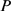 highest confidence regulators from 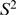, i.e. the 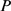 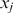
's corresponding to the highest strictly-positive 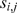
's, where 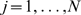. We denote 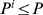 to represent the actual number of regulators chosen, as in general a target gene, 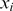, may have less than 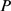 regulators with 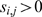. We denote this 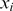 specific subset of likely regulators as 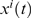.

We use Inferelator 1.0 to learn a sparse ODE model for each 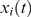 as a function of 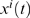 by assuming that the time evolution in the 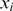
's is governed by
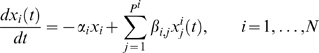
(28)which is exactly (8) with the modification that we only consider a subset of regulators (high confidence ones) for each target gene.

Least Angle Regression (LARS) [32] is used to efficiently implement an 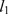 constraint [31] on 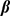, which minimize the following objective function, amounting to a least-square estimate based on the ODE (28):
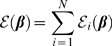
(29)where
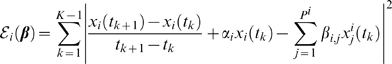
(30)under an 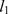-norm penalty on regression coefficients,
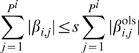
(31)where 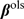 is the over-fit ordinary least-squares estimate (i.e. the minimizer of (30) with no penalty), and 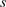 is a number between 0 and 1 referred to as the shrinkage parameter; setting 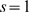 corresponds to ordinary least-square regression. Note that, as before, we use steady-state observations by setting the first term under the summation on the right-hand-side of (30) to zero.

Ten fold cross validation is used to select the minimum value of 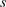 that results in models with good generalization, i.e. good predictive performance on new data. Each resulting model is then an ODE describing the time evolution of 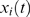. The full set of models, one for each target, constitutes the full network model. For Inferelator 1.0 we have assumed that each target gene has no more than ten regulators, i.e. we have chosen 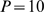 corresponding to 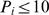 in (28). This assumption turned out to be very wrong for one out of the five 100-gene networks (Yeast3), which had genes that were regulated by as many as 24 regulators.

To produce the ranks required by the challenge we combine the Inferelator 1.0 model weights (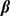) with the mixed-CLR measures of confidence (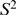) using a simple heuristic designed to give each method roughly equal influence. We describe this heuristic in the following two sub-sections.

#### Step 3.b: Converting Inferelator 1.0 Weights into Confidence Scores

Regulatory interactions that were supported by mixed-CLR and not filtered out all have corresponding confidence scores 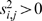, in 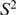. The previous, Inferelator 1.0, step gave us a sparse matrix, 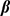, with a small number of entries 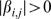, chosen from the regulatory interactions with 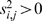.

To ensure that Inferelator 1.0 confidence scores are on equal footing with the previous confidence scores, stored in 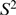, we first assigned all Inferelator 1.0 weights to 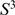, i.e. 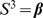, and then replaced the non-zero values (weights) in 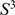 with a corresponding confidence scores of equal rank in 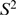. For example, the regulatory interaction with the highest absolute value, 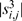 in 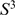, was assigned the highest value from 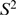, while the interaction with the second highest absolute value in 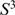, was given the second highest value from 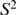. We continued in such a way until we assign a confidence score from 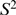 to each interaction in 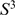 that had a non-zero weight.

#### Step 3.c: Combining Results from Mixed-CLR and Inferelator 1.0 to Produce Final Ranks

We store our final confidence scores for regulatory interactions that were supported by mixed-CLR and Inferelator 1.0, in 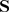, with every entry 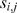 equal to
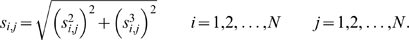
(32)Note that most confidence scores in 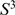 equal zero (the Inferelator 1.0 weight was zero) and thus have no effect on the final confidence scores. This step can be considered as re-organization (pushing up the ranking list) of regulatory interactions with 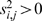 that also had an Inferelator 1.0 model weight 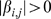.

The regulatory interactions scored in 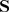, when ranked from high to low, represents our final ranking for the regulatory interaction list. It is given that auto-regulatory interactions do not exist in the DREAM3 challenge networks, thus we have not considered auto regulatory interactions, 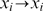, for all 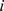.

We have implemented all the steps in our pipeline using the R statistical language [40]. Code is freely available from the authors upon request.

### Comparing Results to Inferelator 1.0 alone

Inferelator 1.0 as previously described in [25] used a dynamic correlation matrix, similar to 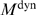 (16) (with the only difference being that dynamic correlation was used as a measure of similarity instead of dynamic mutual information), to initially choose high confidence regulators. In order to compare performance of Inferelator 1.0 alone to the pipeline described above, we also computed the correlation between every dynamic pair, 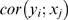 (see equations (14) and (15)), and stored the values in the form of a 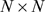 matrix, 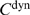. We then performed step 3.a using 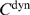 instead of 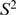 and ranked chosen regulatory interactions, i.e. regulatory interactions corresponding to 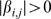, based on absolute value weights. The resulting ranked list was used to evaluate the performance of Inferelator 1.0 alone.

### Judging Performance

After a network inference method suggests potential regulatory interactions, validation of these interactions typically requires significant effort (often requiring the coordination of multiple experiments). Hence, a regulatory network inference method should ideally produce a small number of false positives (FP) even at the expense of a higher false negative (FN) rate. When testing such a method, the performance metric should be sensitive to the method's ability to avoid FPs. Therefore, throughout this section we used area-under-curve of precision (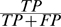) vs. recall (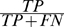) plot, where TP stands for true positives, as a measure of performance, since it degrades quickly with FPs.

## Results

### Mixed-CLR and Inferelator 1.0 Proved Complimentary, Outperforming Other Methods and Combinations of Methods

**Figure 2:**
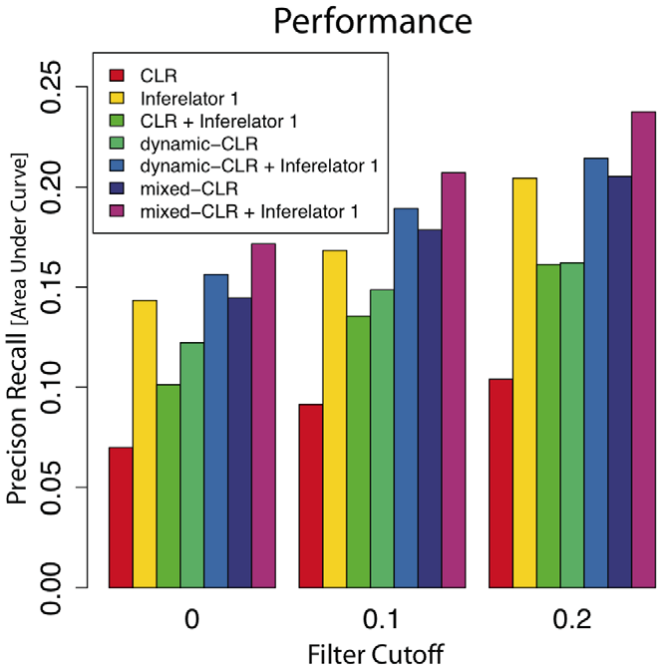
Mean area-under precision vs. recall curves for DREAM3 five 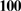-gene networks. We evaluated the performance of Inferelator 1.0 and three different versions of CLR—namely: original-CLR (CLR), dynamic-CLR, and mixed-CLR—with or without Inferelator 1.0, at three levels of knock-out filtration, 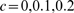. To make DREAM3 predictions we used mixed-CLR with Inferelator 1.0 (with filtration cutoff 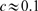), resulting in area-under precision vs. recall curve of 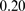 (p-value, 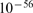), and area-under receiver operating characteristic curve of 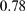 (p-value, 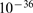). We show that the pipeline we used to make DREAM3 predictions produced optimal performance, compared to other tested CLR/Inferelator 1.0 combinations. Error bars for methods involving Inferelator 1.0 (variability due to cross validation) are approximately within 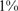 of Precision vs. Recall area-under-curve values and are thus not shown.

We used the DREAM2 50-gene data for testing our pipeline prior to the DREAM3 100-gene challenge. On both this pre-competition data and the actual DREAM3 data, Mixed-CLR with Inferelator 1.0 outperformed other potential pipelines we evaluated, and was thus the method we initially used for the DREAM3 competition. From Figure 2 we can see that: 1) mixed-CLR outperformed dynamic-CLR and CLR, regardless of filtration cutoff (for the DREAM3 networks we used a mean filtration cutoff of 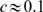, so as to filter approximately one third of all regulatory interactions for each network); 2) our simple knock-out filtration step boosted performance of any method combination we tested but did not alter the performance ranks of the methods tested for any cutoff value; and 3) Mixed-CLR and Inferelator 1.0 are complimentary, providing superior performance when compared to each method alone.

The same trend, in which mixed-CLR coupled with Inferelator 1.0 outperforms the other evaluated method combinations for a large range of tested filtration cutoffs, holds for DREAM3 50-gene networks (for which our method ranked 4th out of 27) and 10-gene networks (for which our method ranked 5th out of 29) (data not shown). As for the DREAM3 50-gene networks, our pipeline did not outperform the DREAM2 50-gene challenge best performers [27]–[29], [42] (data not shown). Note that our method is based in-part on computing z-scores. As network (or system) size decreases, our estimates for underlying probability distributions decreases as well, making our z-score estimates crude. This perhaps explains the decline in performance (relative to other participating methods in DREAM3) for the smaller networks in this challenge.

### For DREAM3 100-Gene Networks, Knock Out Observations Contributed Most to Performance

**Figure 3:**
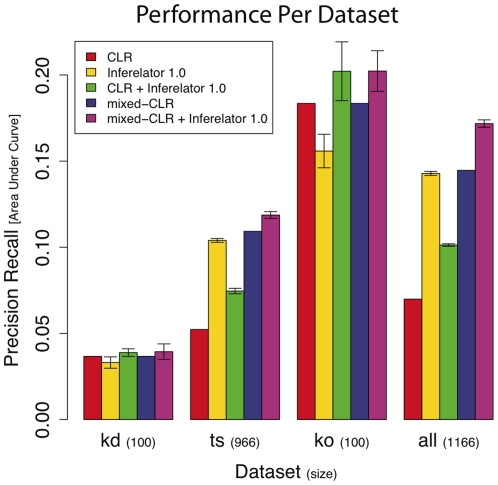
Performance as a function of data set used. We evaluated the contribution of each data set (namely: knock-down (‘**kd**’), time-series (‘**ts**’), knock-out (‘**ko**’), and all three combined (‘**all**’)) to performance of CLR, mixed-CLR, Inferelator 1.0, and CLR or mixed-CLR with Inferelator 1.0 (no filtration was used, 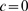). Note, mixed-CLR is a generalization of CLR that takes advantage of time-series data, when time-series data is not used (i.e. for ‘kd’ and ‘ko’) the two are equivalent. For all tested methods ‘ko’ data contributes the most to performance (followed by ‘ts’ and ‘kd’ data respectively). The inclusion of a dynamical model allowed mixed-CLR and Inferelator 1.0 to take advantage of ‘ts’ data (compare to CLR above ‘ts’ and ‘all’ data partitions). Mixed-CLR and Inferelator 1.0 are complimentary, as evidenced by the improvement in performance when the two methods are combined. For ‘ts’, ‘ko’, and ‘all’ data partitions, mixed-CLR with Inferelator 1.0, the method we used to make predictions for DREAM3, gave optimal performance. Error bars for methods involving Inferelator 1.0 are drawn at one standard deviation (estimated from ten Inferelator 1.0 runs).

One important question that the DREAM initiative aims to answer is what data sets are most useful for characterizing regulatory network. We compared the performance of five methods (CLR, mixed-CLR, Inferelator 1.0, and mixed-CLR or CLR with Inferelator 1.0) over four partitions of the full range of provided experiments, namely: knock-down, knock-out, time-series, and the former three combined. From Figure 3 we can see that: 1) the dynamical methods, mixed-CLR and Inferelator 1.0 were more powerful at utilizing time-series observations than the static method CLR; 2) for all dataset partitions tested, mixed-CLR and Inferelator 1.0 proved complimentary and had optimal performance; and 3) for all tested methods, knock-out data (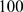 observations) was most instrumental for learning the regulatory networks, followed by time-series and knock-down data (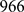 and 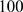 observations, respectively).

### Inferelator 1.0 and Use of Knock-out Information Effectively Resolved Causation

Determining causation (the directionality of regulatory interactions) is one of the tougher problems to solve when inferring regulatory networks. In practice, *a priori* knowledge is often used to suggest which genes are regulating a given target or target set (for example knowing that one gene codes for an enzyme and one for a transcription factor gives us the ability to resolve directionality). However solving for the directionality between pairs of regulators remains a critical challenge. It could be argued, for example, that determining regulatory interactions between pairs of regulators is a more important problem than resolving other regulatory interactions, as interactions between regulators are key to the cell's ability to process and integrate information.

**Figure 4:**
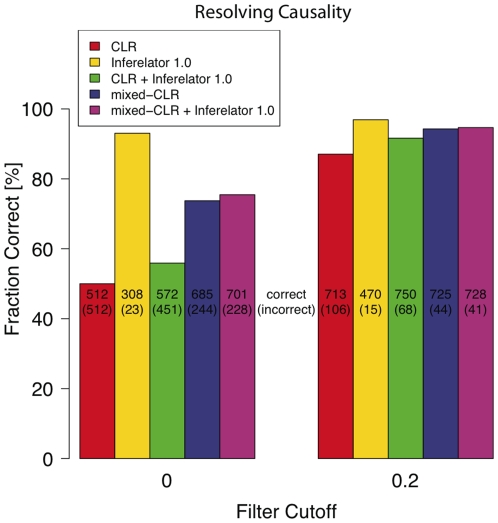
Resolving causation of regulatory interactions. We present the relative merit of five methods, with and without knock-out filtration, to resolve causation (i.e. directionality of regulatory interactions). For each method we computed the fraction of correctly resolved true regulatory interactions (true positives, TPs) out of the total number of TPs the method had identified. We define a TP interaction, 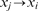, as correctly resolved, if its score, 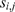 (according to each method or method combination), was bigger than the confidence score of the reverse (false) regulatory interaction, 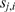. The original CLR method without filtration results in symmetric confidence scores, 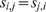, and thus cannot resolve causation (fraction correct = 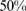). In each bar plot we report the absolute number of correctly (incorrectly) resolved interactions. We show that, without filtration, Inferelator 1.0 has the most power at resolving causation (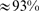 correct), and that for all methods knock-out filtration helps resolve causation. For Inferelator 1.0 filtration helps recover more TPs. Error bars for methods involving Inferelator 1.0 are less than 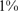 and are not shown.

We compared the relative merit of five methods (CLR, mixed-CLR, Inferelator 1.0, and mixed-CLR or CLR with Inferelator 1.0) with or without knock-out filtration to determine causation. From Figure 4 we can see that: 1) Out of the five methods, Inferelator 1.0 best resolved causation (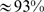); 2) mixed-CLR had some power at resolving causation when compared to the static version of the algorithm; and 3) removal of unlikely regulatory interactions based on the knock-out filtration, was very useful for resolving causation and complimentary to the other methods we tested.

### Performance Degrades with Increasing Network In-Degree

**Figure 5:**
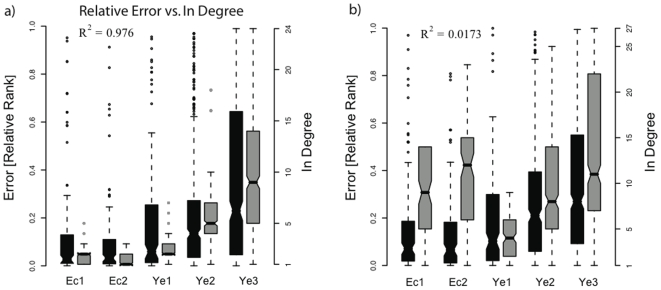
Error as a function of gene in degrees and gene out degrees. Here we evaluate the performance of mixed-CLR, filtration cutoff of 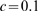, and Inferelator 1.0—the pipeline we applied to make DREAM3 predictions. Box plots for error distributions for each of the five predicted networks are shown in black in both panels, gray box plots show in-degree and out-degree distributions for **a.** and **b.** respectively. We estimated error in the following manner: Denote by 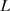 the total number of possible regulatory interactions, and by 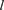 the rank we gave to a regulatory interaction, 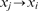, the relative rank (error) of 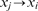 is defined to be 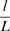. **a**) Median relative rank (Error) increases as the networks' median in-degree increases (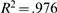). **b**) Median relative rank (Error) is not correlated with median out-degree (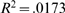).

Biological regulatory networks are typically sparse, i.e. they have a relatively small number of regulatory edges when compared to the total number of possible edges. Network sparsity is commonly used to glean at what the dynamic complexity of that network would be if it could be simulated or observed (where the more sparse a network is, the simpler its dynamic behaviour becomes). Network sparsity in turn can be separated into two more detailed measures: network in-degree distribution, derived from the distribution of regulatory edges entering each target gene, and network out-degree distribution, derived from the distribution of regulatory edges leaving each regulator. Each distribution when summed equals to the number of regulatory edges in the network. We find that, as expected, our method's median error increases with genes median in-degree (see Figure 5) (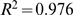), i.e. performance drops for targets under the control of many regulators, but interestingly is not correlated to median out-degree (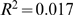), i.e. performance does not drop for regulators controlling many target genes.

### For DREAM3 100-Gene Networks, Mixed-CLR Did Not Effectively Correct for Background

**Figure 6:**
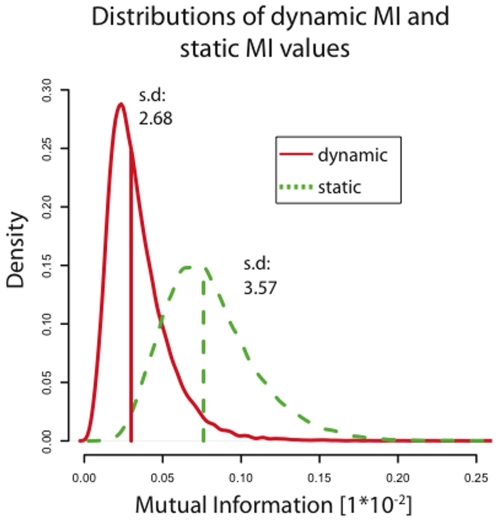
Distributions of static vs. dynamic mutual information values. We computed static and dynamic Mutual Information (MI) values for every possible regulatory interaction. Vertical lines represent distribution means. We present the combined probability densities for the five 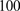-gene networks. We show that: 1) both dynamic and static MI densities are right skewed, consistent with the assumption that MI values of true positives would be higher than MI values of true negatives; 2) the standard deviations for static MI z-scores, 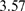, is larger than for dynamic MI z-scores, 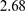, possibly making it easier to recover TPs from the dynamic MI z-scores; and 3) most dynamic MI values are smaller than the mean of the static MI values; this shift confounds mixed-CLR. Note that both static- and dynamic-MI values were estimated from the same number of observations, using the same number of bins. Thus, dataset size or bin number differences do not explain the shift in distributions.

One unexpected problem with mixed-CLR (that the DREAM3 challenge revealed) is that we have no guarantee that static and dynamic MI values will be in the same range for a given data set (which we assumed when constructing mixed-CLR). Indeed, from Figure 6 we can see that the majority of dynamic-MI values were below the mean static MI value. Since for background correction mixed-CLR computed the positive z-score of each regulatory interaction's dynamic MI value, assuming it was taken from the distribution of static MI values, most of these z-scores ended up being zero. Thus, for DREAM3 100-gene networks mixed-CLR in practice was the result of determining z-scores for each regulatory interaction based on the dynamic MI values alone (dynamic MI z-scores).

Also, we can see from Figure 6 that the dynamic MI distribution had a smaller standard deviation (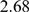) than the static MI distribution (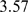), possibly making it easier to resolve true regulatory interactions from false regulatory interactions.

### Dynamic MI Identified True Regulatory Interactions Better Than Static MI

**Figure 7:**
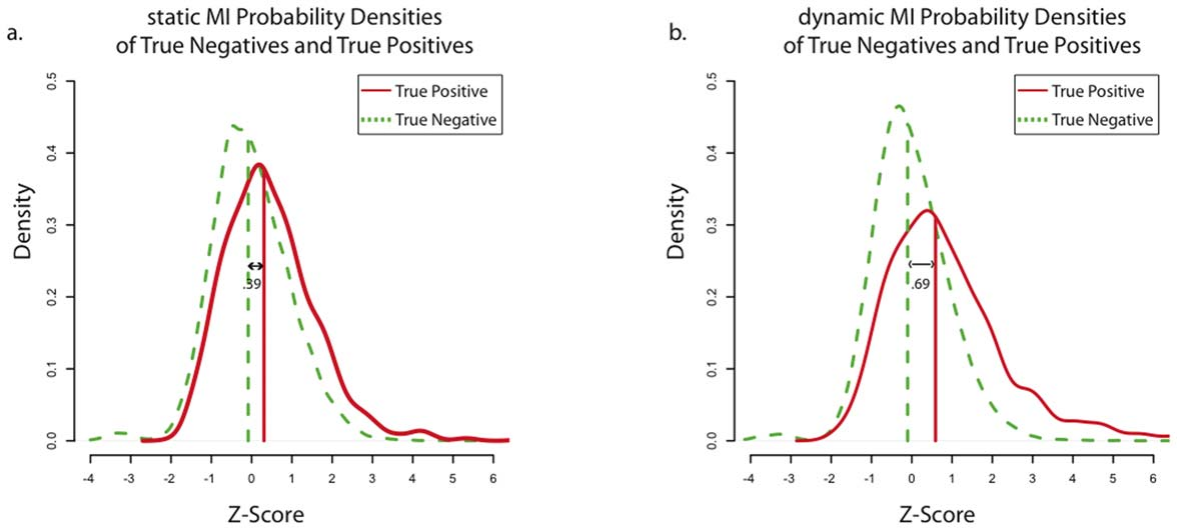
Probability densities of static and dynamic mutual information values for true positive and true negative regulatory interactions. We computed static and dynamic Mutual Information (MI) values for every possible regulatory interaction for all five 100-gene networks. For both static and dynamic MI values, we computed z-scores for true regulatory interactions (true positives, TPs) and false regulatory interactions (true negatives, TNs). We present the static (**a.**) and dynamic (**b.**) z-scores densities (combined over the five 100-gene networks) for TPs (red) and TNs (green). Vertical lines represent median z-scores. We show that TPs are better separated from TNs by the dynamic MI z-scores, consistent with the improved performance of mixed- and dynamic-CLR.

We hypothesized that dynamic-MI will decrease false statistical dependencies between gene pairs (i.e. dependencies that are not due to direct regulatory interactions), assisting in the identification of true regulatory interactions. To test this hypothesis we computed MI between the expression levels of every gene pair (static MI), and between every pair of dynamic response and explanatory-variable (dynamic MI). For both static and dynamic MI values, we computed a z-scores for each true regulatory interaction (true positive, TP) and false regulatory interaction (true negative, TN) by assuming its MI value is taken from the distribution of MI values involving the target in that interaction, i.e. the first z-score from dynamic-CLR or mixed-CLR. Indeed, from Figure 7 we can see that TPs are better separated from TNs by dynamic MI z-scores than by static MI z-scores.

### Top Ranked Predictions are Largely Correct

As mentioned previously, in biology it is desired that methods have high precision even in the expense of recall (completeness). Here we take a look at precision for several recall values ranging from low to high recall (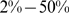). We show in table 1 the performance for the method's best predicted network, Ecoli 2, and in table 2 the performance for the method's worst predicted network, Yeast 3. Our full pipeline produced good precision results, especially for the lower recall values. The boost in performance by Inferelator 1.0, that seems to be mostly apparent in the lower recall values, is probably confounded by an overly strong sparsity penalty at higher recall values for more complex models, i.e. Inferelator 1.0 identifies interactions with a high precision, but it seems to be too parsimonious to identify a substantial portion of the true regulatory interactions for high in-degree networks, e.g. the Yeast 3 network (where target genes exhibit in-degrees exceeding 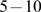, see Figure 5). Nevertheless our high accuracy in these low-recall settings is well matched to typical biological laboratory settings.

**Table 1:** Network 2 (E.coli-2): Methods precision for low-to-high recall values.

	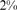	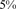	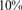	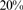	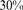	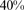	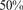
mixed-CLR+Inferelator 1.0	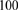	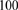	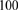	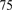	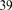	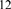	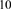
mixed-CLR	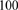	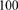	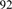	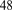	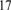	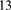	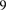
dynamic-CLR+Inferelator 1.0	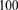	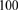	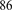	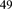	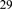	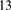	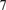
dynamic-CLR	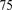	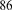	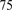	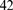	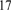	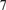	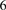
CLR+Inferelator 1.0	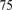	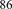	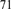	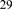	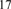	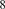	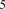
Inferelator 1.0	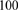	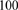	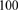	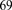	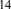	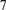	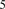
CLR	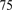	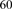	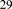	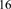	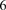	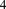	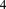

In this table we present a more detailed view of performance for our method's best predicted network (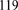 total regulatory interactions with up to 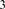 regulators controlling each gene). The table inline method precision [%] at varying degrees of completeness (recall [%]).

**Table 2:** Network 5 (Yeast-3): Methods precision for low-to-high recall values.

	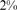	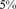	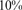	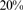	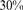	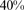	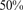
mixed-CLR+Inferelator 1.0	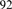	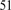	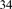	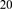	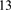	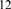	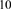
mixed-CLR	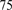	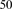	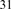	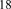	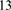	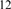	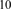
dynamic-CLR+Inferelator 1.0	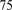	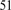	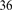	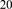	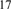	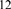	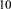
dynamic-CLR	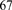	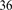	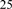	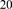	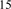	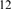	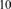
CLR+Inferelator 1.0	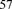	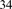	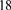	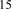	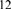	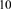	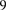
Inferelator 1.0	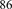	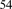	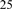	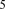	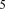	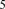	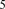
CLR	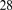	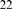	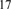	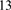	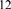	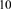	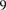

In this table we present a more detailed view of performance for our method's poorest predicted network (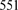 total regulatory interactions with up to 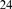 regulators controlling each gene). The table inline method precision [%] at varying degrees of completeness (recall [%]).

## Discussion

We have shown that explicitly modeling dynamics using a simple ODE model increases the ability of our pipeline to identify true regulatory interactions (when compared to a static model), and help resolve the directionality of these interactions. Specifically, analysis of our performance on the DREAM3 100-gene networks show that: 1) the full pipeline (mixed-CLR followed by, knock-out filtration and Inferelator 1.0) outperformed other tested combinations of dynamic and static methods (Figure 2); 2) knock out data was instrumental for learning regulatory interactions (Figure 3); 3) Inferelator 1.0 was instrumental for resolving regulatory causation (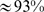 of identified regulatory interactions were correctly resolved, Figure 4). 4) mixed-CLR and Inferelator 1.0 proved complimentary (Figure 2 and 3); and 5) dynamic MI values (mixed-CLR) separated true regulatory interactions from false, but otherwise dependent, pair-wise interactions better than static MI values (Figure 7).

We observed a drop in performance as the median in-degree of a network increases (Figure 5.a). This is to be expected and could be due to many reasons, including: 1) the dynamic behavior of a target gene becomes more condition-dependent as the number of regulators increases, fragmenting the data set among distinct conditions, and making it harder to resolve regulatory interactions from expression data no matter the method used (dynamic or static). This is supported by the observation that any method combination we have tested under-performed on the high in-degree networks (e.g. table 2), compared to low in-degree networks (e.g. table 1); 2) the model we have used was too simple to describe the dynamic behaviour of high in-degree target genes; 3) with Inferelator 1.0 we have imposed an 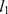 constraint on model weights (i.e. a constraint on in-degree) that may have been too restrictive for high in-degree target genes; and 4) with this use of the Inferelator 1.0 we enforced a strict ten predictor cutoff that proved too stringent for two of the 100-gene networks, which had a significant number of target genes under the control of more than ten regulators.

Interestingly, we have not observed a similar drop in performance as networks median out-degree increased (Figure 5.b). In principle, as a networks' median out degree increases one expects that the number of indirect regulatory interactions (mediated through regulators of regulators) will increase, and with it the underlying complexity of the system's dynamic behaviour. However, there may be many reasons why this was not observed, including: 1) a change in a regulator's mRNA, followed by a corresponding change in a target gene's mRNA requires a time delay (note that for the DREAM3 *in silico* challenges mRNA and protein levels were modeled, albeit observations were only given for mRNA). This time delay will increase for indirect regulatory interactions. Since our method uses consecutive observations (here sampled every 20 minutes) a change in an indirect regulator's mRNA levels may not have the time to effectively propagate to its indirect targets. In other words, the observations were sampled finely enough to make direct regulatory interactions resolvable from indirect ones; 2) our approach was centered around the target gene; we modeled the change in rate of expression of each gene separately, i.e. we assumed an un-coupled system of ODEs. Thus our model complexity is largely determined by the number of regulators a gene has (in-degree), but not by number of targets a regulator has (out-degree); and 3) two (out of five) of the networks, the ones responsible for the observed lack of correlation between out-degree and performance, were based on *E.coli*'s topology. There is evidence that for *E.coli* the number of indirect regulatory interactions (and thus complexity) is much smaller than expected by its out-degree distribution [43], [44], and that its transcriptional network has primarily a feedforward structure, resulting in less complex dynamics due to the relative lack of feedbackward loops that would otherwise keep information propagating in the network (and thus increase complexity) [43], [44].

We learned that the Inferelator 1.0 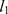-norm regularizer (LARS [31], [32]) proved to be too parsimonious for the two most complex (in terms of target in-degree) 100-gene networks, leaving many true regulatory interactions out of the model. One limitation of using an 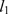 constraint is that in cases where several explanatory variables are correlated (or anti-correlated), the procedure will tend to pick either one of them or none, potentially leading to overly sparse models. This suggests that using a method which is more robust to the “one or none” problem, such as the elastic-net [45] (an 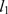 and 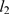 norm constrained regression), will improve performance.

We were encouraged to see that even a very simple dynamical model was able to significantly increase performance (compared to static model) at identifying true regulatory interactions and resolving their causation. Moreover, the two dynamic methods mixed-CLR and Inferelator 1.0 proved complimentary.

Knock-out observations were instrumental for characterizing the DREAM3 100-gene regulatory networks (Figure 3). This is in line with our observation that even the crude filter we used (based in part on knock-out data) to remove the least likely regulatory interactions proved very effective in identifying true regulatory interactions (Figure 2) and resolving their direction (Figure 4). Importantly, when knock-out data was used alone, all tested methods achieved optimal performance (Figure 3). Furthermore, only 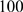 knock-out observations were needed, compared to the 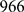 provided time-series observations (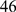 time-series experiments, each containing 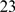 observations). In our previous works we assumed that Inferelator 1.0 would implicitly use genetic information (e.g. knock-out data) by incorporating the steady-state data into the learning procedure, hence not requiring explicit constraints to be derived from genetic perturbations. The DREAM3 results suggest that we need to develop better explicit methods to incorporate constraints from such genetic perturbations into Inferelator 1.0 or similar methods. However, it is typically not possible to obtain knock-out information for each gene in such a comprehensive manner. Even when knock-out information can be obtained, the knocked out gene may not be active under the “wild type” conditions, thus not revealing any regulatory information. Therefore, it will prove helpful to also incorporate other types of constraints, for example constraints derived from TF-DNA binding experiments such as ChIP-chip [46] and ChIP-seq [47], [48].

To conclude, the pipeline we have described here was developed with the aim of producing a sorted, enriched subset of true direct regulatory interactions. We find that our full pipeline was able to find a significant fraction of the true positive regulatory interactions. We also find that our top ranked predictions have very low error rate, suggesting that our method is useful in the context of an active genomics consortia, where network models are improved in an iterative manner: highly ranked predictions of target-gene interactions are validated with new data collection, causing the generative model to be re-updated, allowing for new predictions and validation, etc.
